# Urinary MicroRNA-10a and MicroRNA-30d Serve as Novel, Sensitive and Specific Biomarkers for Kidney Injury

**DOI:** 10.1371/journal.pone.0051140

**Published:** 2012-12-13

**Authors:** Nan Wang, Yang Zhou, Lei Jiang, Donghai Li, Junwei Yang, Chen-Yu Zhang, Ke Zen

**Affiliations:** 1 Jiangsu Engineering Research Center for MicroRNA Biology and Biotechnology, School of Life Sciences, Nanjing University, Jiangsu, China; 2 Research Center of Nephrology, Second Affiliated Hospital of Nanjing Medical University, Nanjing, Jiangsu, China; National Institutes of Health, United States of America

## Abstract

The steadily increasing incidence of kidney injury is a significant threat to human health. The current tools available for the early detection of kidney injury, however, have limited sensitivity or specificity. Thus, the development of novel biomarkers to detect early kidney injury is of high importance. Employing mouse renal ischemia-reperfusion and streptozotocin (STZ)-induced renal injury as acute and chronic kidney injury model, respectively, we assessed the alteration of microRNA (miRNA) in mouse urine, serum and kidney tissue by TaqMan probe-based qRT-PCR assay. Our results demonstrated that kidney-enriched microRNA-10a (miR-10a) and microRNA-30d (miR-30d) were readily detected in mouse urine and the levels of urinary miR-10a and miR-30d were positively correlated with the degree of kidney injury induced by renal ischemia-reperfusion or STZ diabetes. In contrast, no such alteration of miR-10a and miR-30d levels was observed in mouse serum after kidney injury. Compared with the blood urea nitrogen (BUN) assay, the test for urinary miR-10a and miR-30d levels was more sensitive for the detection of acute kidney injury. Furthermore, the substantial elevation of the urinary miR-10a and miR-30d levels was also observed in focal segmental glomerulosclerosis (FSGS) patients compared to healthy donors. In conclusion, the present study collectively demonstrates that urinary miR-10a and miR-30d represent a novel noninvasive, sensitive, specific and potentially high-throughput method for detecting renal injury.

## Introduction

The incidence of kidney injuries, including diabetic nephropathy and drug-induced side effects, is steadily increasing worldwide [1]. Acute kidney injury is considered to be an important risk factor for progression to end-stage renal disease [2]. The early detection of kidney injury would make it possible to diagnosis acute kidney injury in a timely manner, but such detection remains a difficult task, due to the lack of reliable biomarkers. Current metrics for renal function, such as blood urea nitrogen (BUN), lack the sensitivity and/or specificity to adequately detect kidney damage at early stages of the injury [3], [4]. Recently, kidney injury molecule-1 (Kim-1) was identified as a highly sensitive and specific urinary biomarker for kidney injury [5]. Subsequent preclinical studies also showed that Kim-1 is a promising early diagnostic biomarker for monitoring acute kidney tubular necrosis [6]. Whether Kim-1 can serve as a suitable biomarker for various types of kidney injuries, however, requires further investigation using a large number of different renal injury samples.

MicroRNAs (miRNAs) are ∼22-nt long noncoding RNA molecules that have emerged as a new class of gene regulators at the posttranscriptional level. It is widely believed that miRNAs regulate nearly 30% of protein-coding genes and are involved in almost every aspect of developmental, pathogenic and tumorigenesis processes. Accumulating evidence demonstrates that miRNAs serve as mediators in chronic kidney disease [7]. Studies of conditional dicer-knockout mice [8], [9] revealed critical roles for miRNAs in regulating kidney development and maintaining the structural and functional integrity of the renal collecting system and the glomerular barrier. The determination of miRNA expression profiles in normal kidneys and their constituent cells has suggested that specific miRNAs may serve special functional roles in these cells [9]. Kato and co-workers [10] reported on the expression and function of miR-192 in diabetic kidney glomeruli. Employing mouse renal ischemia reperfusion injury (IRI) model, Godwin et al. [11] identified a miRNA signature in kidney tissue that was tightly correlated to renal IRI. Recently, studies by our group [12], [13] and others [14]–[19] have demonstrated that miRNAs can be released by cells and tissues into circulation and that these circulating miRNAs in the serum, plasma, urine, and other body fluids are stable and can serve as noninvasive biomarkers for various diseases and tissue injuries. When studying the serum and urinary levels of the miR-200 family, miR-205 and miR-192 in patients with systemic lupus erythematosus (SLE), Wang et al. [20] found that the levels of most of those miRNAs from patients were lower than the levels of controls, suggesting that miRNA may take part in the pathogenesis of SLE and that miRNAs in the urine and serum could be biomarkers for SLE.

In the present study, we hypothesized that the levels of specific circulating miRNA species could be used to detect and monitor the pathological development associated with kidney injuries. Using different mouse renal injury models, we reported that miR-10a and miR-30d were readily detected in urine and that their levels specifically correlated with mouse kidney injury induced by renal ischemia-reperfusion or STZ treatment. The elevation of the urinary levels of miR-10a and miR-30d was also confirmed in urine samples from patients with focal segmental glomerulosclerosis (FSGS).

## Materials and Methods

### Ethics Statement

All animal experimental procedures were carried out in accordance with the National Institutes of Health Guide for the Care and Use of Laboratory Animals and were approved by the Animal Care Committee of Nanjing University (Nanjing, China).

### Mouse renal ischemia-reperfusion injury (IRI)

Male C57BL/6J mice (6–8 weeks old, 22–25 g) were assigned into three groups with ten mice per group: the sham, unilateral and bilateral ischemia-reperfusion (I/R) groups. Renal I/R was performed according to an established procedure [21]. Briefly, mice were under general anesthesia with 0.4–0.5 g/kg chloral hydrate and kept on a heating pad to maintain the body temperature at 37°C. After a midline abdominal incision, the renal pedicles were exposed by blunt dissection, and a microvascular clamp was applied for 40 minutes. The kidneys were observed after removal of the clamp(s) to assess reperfusion. After reperfusion, ∼0.8 ml of pre-warmed saline was placed in the peritoneal cavity, and the abdomen was closed in layers. Sham control mice were subjected to an identical operation without renal pedicle clamping. Mouse urine was collected using a metabolic cage at 18∼24 h and 30∼36 h after reperfusion, respectively. Mice were sacrificed at 24 and 36 h after reperfusion and blood samples were collected and the kidneys were removed. Part of the kidney tissue was fixed in 10% phosphate-buffered formalin followed by paraffin embedding for histological study.

### Renal function determination

Renal function was monitored by measuring the BUN concentration using analytical kits according to the protocols specified by the manufacturer (Rongsheng Biotech, Shanghai, China). Mouse blood samples were collected when mice were sacrificed. Samples were centrifuged to separate plasma.

### Histological staining

Renal histology was examined by hematoxylin/eosin staining. Briefly, kidneys were collected and fixed in 10% phosphate-buffered formalin at 4°C overnight. The kidneys were subsequently paraffin embedded and sectioned (4 µm). Sections were stained with hematoxylin/eosin for general histology. Images of representative fields were recorded.

### RNA extraction

Total RNA was extracted from 100 µL samples (serum or urine). In brief, 100 µL sample was mixed with 300 µL diethylpyrocarbonate-treated water, 200 µL acid phenol, 200 µL chloroform. The mixture was vortex-mixed vigorously and centrifuged at 12000 g for 15 min at room temperature. After phase separation, the aqueous layer (∼400 µL) was mixed with 2 volumes of isopropyl alcohol and 0.1 volumes of 3 mol/L sodium acetate (pH 5.3). This solution was stored at −20°C for 1 h. The RNA pellet was collected by centrifugation at 16000 g for 20 min at 4°C. The resulting RNA pellet was washed once with 750 mL/L ethanol and air-dried for 10 min at room temperature. Finally, the pellet was dissolved in 20 µL of ribonuclease-free water.

### Streptozotocin (STZ)-induced kidney damage

Kidney injury was established in STZ-induced diabetic mice as previously described [22]. Male C57BL/6J mice were divided into two groups (eight mice/per group) after measuring the body weight. Group 1 was intraperitoneally injected with 50 mg/kg of STZ dissolved in 10 mM citrate buffer (pH 4.5) for 5 consecutive days. Group 2 was given an equal volume of citrate buffer. At week 8 post-injection, individual mice were placed in metabolic cages to obtain 24 h urine collections. Blood glucose levels from the tail-vein were measured using the OneTouch Blood Glucose Monitoring System (LifeScan, Milpitas, CA) at 2, 4, 6 and 8 weeks post-injection. The mice were fasted for 4 h before blood glucose measurement. Freshly voided spot urine samples were collected at 1, 4 and 8 weeks post-injection. Urinary albumin was measured by ELISA kits (Bethyl Laboratories, Houston, TX). After mice were sacrificed, kidneys were collected, washed with saline, dried with filter paper and weighed with a precision balance.

### Urine and blood samples from healthy donors and FSGS patients

The present study was approved by the Institutional Review Board of Nanjing University (Nanjing, China) and written informed consent was obtained from each participant. Urine and blood samples (10–15 mL) were collected from 16 healthy donors and 16 FSGS patients (Table 1). Renal function was monitored by measuring BUN, blood pressure, urinary protein, serum creatinine, BUN, uric acid, creatinine clearance, triglyceride and total cholesterol.

**Table 1:** Clinical features of 16 FSGS patients vs 16 normal volunteers.

Clinical features	Normal (Mean ± SEM)	FSGS (Mean ± SEM)
Age (year)	29.1±3.7	26.6±2.9
DD (month)	-	4.78±0.81
BP (mmHg)	128.5±3.1/79.6±3.3	135.1±1.5/84.5±2.6
Upro (g/24 h)	0.27±0.12	6.86±1.03**
URBC (×10^4^)	2.78±1.06	6.81±2.32*
Scr (mg/dl)	0.86±0.13	0.80±0.04
BUN (mg/dl)	14.6±1.61	15.3±1.3
Ccr (ml/min)	108.7±9.79	90.3±15.83
TG (mmol/l)	1.47±0.63	3.39±0.76*
TC (mmol/l)	4.38±1.64	9.83±1.10*

DD: Duration of the disease; BP: Blood pressure; Upro: Urinary protein; URBC: Urinary red blood cell; Scr: Serum creatinine; BUN: Blood urea nitrogen; Ccr: Creatinine clearance. TG: Triglyceride; TC: Total cholesterol.

* , p<0.05;

** , p<0.01.

### TaqMan probe-based qRT-PCR of miRNAs

Total RNA including miRNA was isolated from various tissues of C57BL/6J mice and mouse or human urine and sera using TRIzol reagent (Invitrogen, CA) as previously described [12], [13]. Stem-loop qRT-PCR assays using TaqMan miRNA probes (Applied Biosystems) were performed [13], [23]. Real-time PCR was performed using a TaqMan PCR kit and an Applied Biosystems 7300 Sequence Detection System. The reactions were incubated in a 96-well plate at 95°C for 10 min followed by 40 cycles of 95°C for 15 sec and 60°C for 1 min. All reactions, including no-template controls, were performed in triplicate. After the reactions, the cycle threshold (C_T_) data were determined using default threshold settings, and the mean C_T_ was determined from the triplicate PCRs. A comparative ΔC_T_ method was used to compare each condition with controls, and values were expressed as 2^−ΔCT^. The relative levels of miRNAs in tissues were normalized to U6, a ubiquitously expressed small nuclear RNA. For quantification of sera and urinary miRNA, an equal amount of the synthetic plant MIR168a was spiked in each serum sample prior to RNA extraction to serve as internal control.

### Statistical analysis

For each experiment, qRT-PCR assays were performed in triplicate. The data shown are represented as means ± SEM for at least three independent experiments. Differences were considered statistically significant at p<0.05, as assessed using Student's *t*-test.

## Results

### Identification of miR-10a and miR-30d as kidney-specific miRNAs

**Figure 1:**
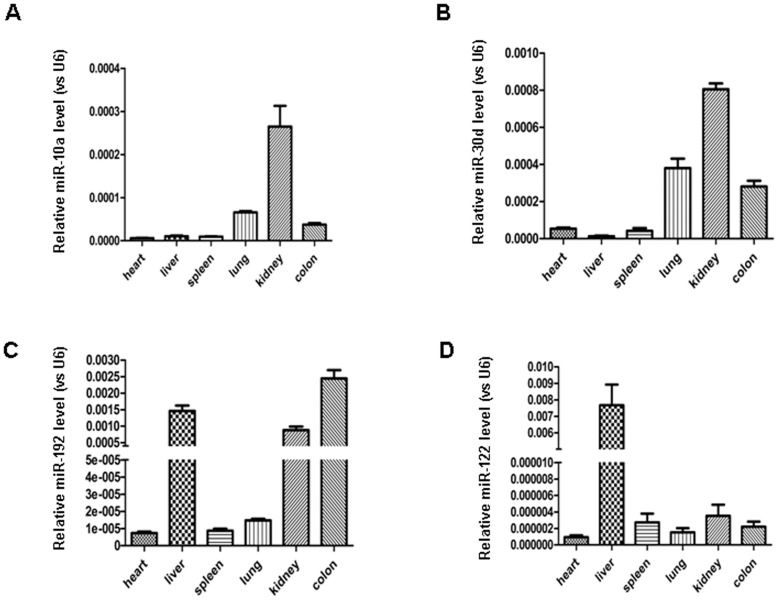
Identification of miR-10a and miR-30d as mouse kidney-enriched miRNAs. RNA from various organs of normal C57BL6 mice was extracted, and the miRNA levels were determined by qRT-PCR assay using U6 as an internal control.

The role of miRNA in kidney function has been illustrated previously by investigators through the specific knockdown of Dicer activity in podocyte cells [8], [9]. Tissue profiling assays further demonstrated that the kidney and its constituent cells contain unique miRNAs, particularly members of the miR-10 and miR-30 families [24] and miR-192 [10]. Depending on the species, the profile of miRNAs in the kidney might be different. Employing Solexa sequencing, we previously analyzed the miRNA expression profile in various mouse tissues, and as shown in Table S1, different tissues have different miRNA profiles. For mouse kidney, after rule out the miRNAs with very low total signal, we found that miR-10a and miR-30d, as well as other miRNAs in miR-1 and miR-30 families, were relatively enriched in kidney tissue. This result was further validated using a TaqMan probe-based qRT-PCR, we detected the expression of miR-10a, miR-30d and miR-192 in various mouse organs: the heart, spleen, kidney, colon and lung. The expression of miR-122, a liver-specific miRNA, served as a control. To compare the expression of a miRNA in various tissues, we normalized the expression level of miRNA against the expression of U6 in the same tissues. As shown in Figure 1, we found that mouse kidneys contained a significantly higher level of miR-10a and miR-30d than did other tissues, confirming that these two miRNAs are kidney specific. Although miR-192 was detected at a high level in the kidney, it was also strongly expressed in the liver and colon. In agreement with a previous report [25], miR-122 was predominantly expressed in mouse liver.

### Elevation of the level of kidney-specific miRNAs in mouse urine after kidney injury

**Figure 2:**
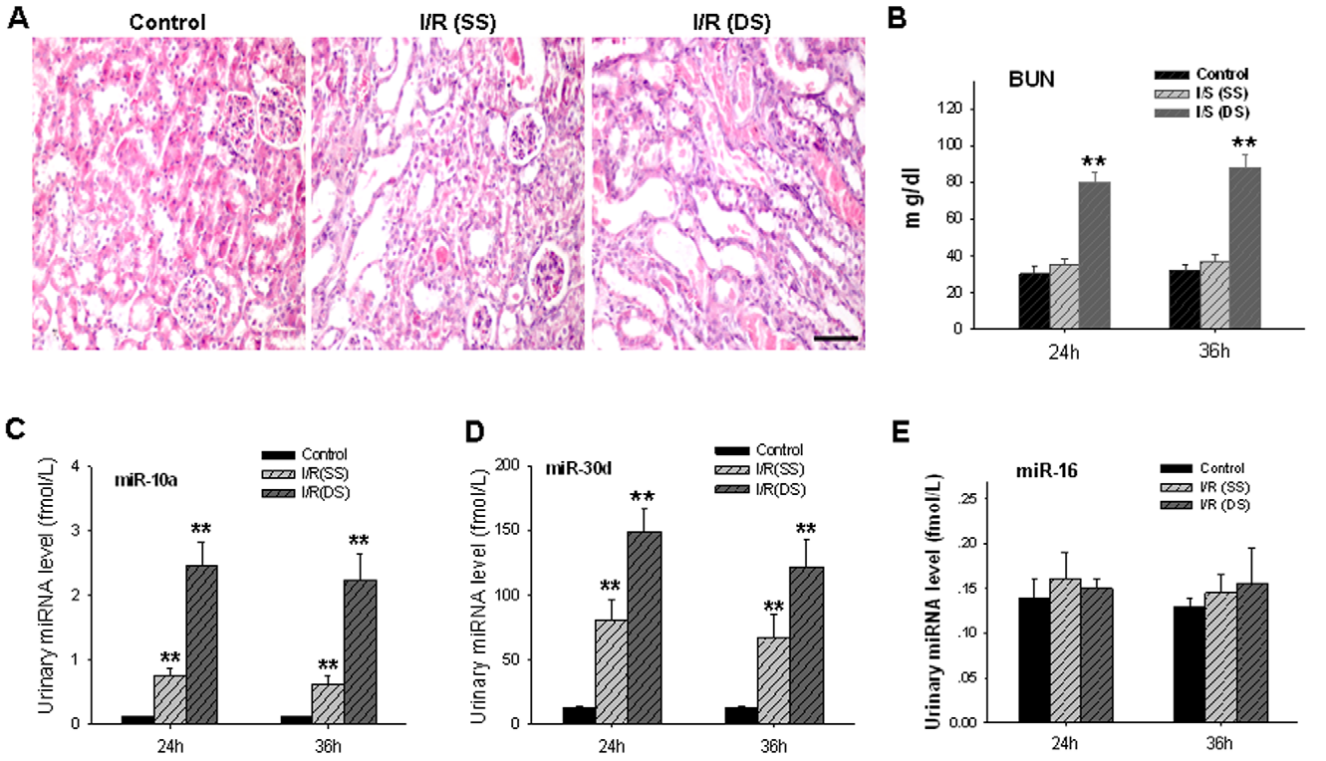
Elevation of the urinary miR-10a and miR-30d levels in mice with renal I/R-mediated injury. **A**, mouse kidney injuries caused by unilateral (SS) or bilateral (DS) I/R. **B**, BUN assay of mouse kidney injury by I/R. Note that the BUN assay was able to detect kidney injury caused by DS I/R but not that caused by SS I/R. **C–D**, significant elevation of the urinary miR-10a (C) and miR-30d (D) levels in mice with either SS I/R or DS I/R. **E**, no alteration of miR-16 in mouse urine after I/R-mediated kidney injury. The data are presented as means ± SEM for six independent experiments; three mice were used in each experiment. *, p<0.05. **, p<0.01.

Based on the tissue distribution of miRNAs determined by qRT-PCR assays, miR-10a and miR-30d were selected as kidney-specific miRNAs for further analysis. Next, we tested whether miR-10a and miR-30d are released into animal urine under normal and injury conditions. In this experiment, we employed unilateral and bilateral renal ischemia/reperfusion (I/R) in mice as a kidney injury model. Urine samples from normal male C57BL/6J mice (6–8 weeks old, 22–25 g) and male C57BL/6J mice with kidney injuries were collected, and absolute levels of miR-10a and miR-30d were assessed. As shown in Figure 2A, unilateral and bilateral renal I/R caused pathological damage to mouse kidneys in various degrees. Compared with the sham control group, transient ischemia caused marked tubular injury, as shown by loss of brush border, tubular dilation, and intratubular cast formation; unilateral and bilateral renal I/R kidney also showed glomerulus atrophy with enlarged capsular spaces and tubule atrophy with compensatory hypertrophy of the adjacent glomeruli. Although no fibrous connective tissue desmoplasia or inflammatory cell infiltration was observed in the injured kidneys, part of the tubule, particularly in bilateral I/R kidneys, exhibited cellular and hyaline cast formation and significant tubular dilatation. Several tubular epithelial cells had papillary hyperplasia and granular degeneration. The kidney injury was also determined using the BUN assay (Figure 2B). In agreement with this, we also detected an increased level of KIM-1 [26] and neutrophil gelatinase-associated lipocalin (NGAL) [27] in I/R kidney (Figure S1). As can be seen, blood urea nitrogen was elevated in mice with bilateral I/R but not the mice with unilateral I/R. Because the tissue section and pathological analysis clearly suggested damage to the mouse kidney under unilateral I/R, the failure to detect the elevation of BUN in the mice with unilateral I/R is indicative of the sensitivity limitation of this method.

Conversely, we found that miRNAs were normally expressed in mouse urine at 0.1–15.0 fmol/L range and were readily detected by the qRT-PCR assay. More importantly, the levels of urinary miR-10a and miR-30d were significantly increased in mice with either unilateral ischemia/reperfusion or bilateral ischemia/reperfusion. Compared with the sham control group, unilateral and bilateral ischemia/reperfusion kidney resulted in 5.5- and 19.5-fold elevations of urinary miR-10a, respectively (Figure 2C), and 5.4- and 10.0-fold elevations of urinary miR-30d, respectively (Figure 2D). The urinary levels of miRNAs that were not specifically expressed in kidneys, such as miR-16, were not altered following either unilateral or bilateral ischemia/reperfusion (Figure 2E). Together, these results strongly suggest that urinary miR-10a and miR-30d could serve as sensitive and specific biomarkers for kidney injury.

Previous studies by our group and others have demonstrated that the alteration of the serum miRNA expression profile can be used as a molecular fingerprint for various diseases [12] and organ injuries [17], [20]. Therefore, we also detected the levels of miR-10a and miR-30d in mouse serum with or without renal I/R. Interestingly, we found that the miR-10a and miR-30d levels in serum were not correlated with kidney injury. As shown in Figure S2, no alteration of miR-10a or miR-30d in mouse serum was observed after unilateral renal I/R. After bilateral renal I/R, the level of miR-30d in serum was still unchanged, while the level of miR-10a was reduced. The elevation of kidney-enriched miR-10a and miR-30d in urine (Figure 2) but not serum (Figure S2) during renal I/R indicated that these miRNAs may be directly correlated with kidney injury.

Next we determined the levels of miR-10a and miR-30d in mouse kidney tissue with or without renal I/R. As shown in Figure S3A, both miR-10a and miR-30d in mouse kidney were significantly reduced during renal I/R. Interestingly, the levels of pre-miR-10a and pre-miR-30d in mouse kidney tissues were not changed (Figure S3B). Moreover, pre-miR-10a and pre-miR-30d were not detected in mouse urine by qRT-PCR (data not shown). These results collectively suggest that elevation of mouse urinary miR-10a and miR-30d during renal I/R is likely due to the release of mature miR-10a and miR-30d from mouse kidney tissue.

**Figure 3:**
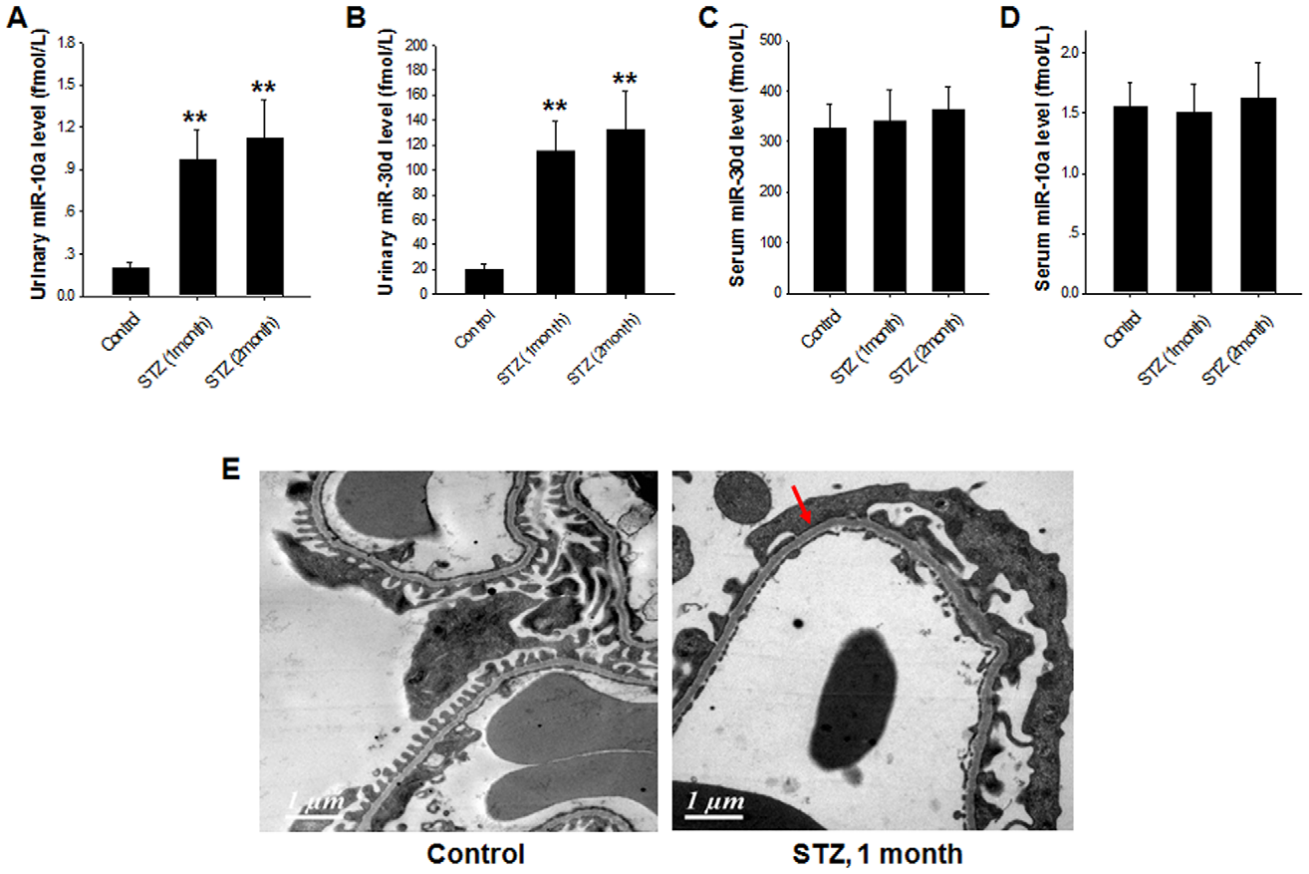
Elevation of the urinary miR-10a and miR-30d levels but not serum miR-10a and miR-30d in mice with STZ diabetes-associated kidney injury. Note that the levels of urinary miR-10a (**A**) and miR-30d (**B**) were significantly increased in mice with STZ diabetes-induced kidney injury, whereas the levels of serum miR-10a (**C**) and miR-30d (**D**) were not altered. **E**, a represented image of electron microscopy showing that mice with 1 month STZ treatment have segmental podocyte foot process effacement (arrow). The data are presented as means ± SEM for six independent experiments; three mice were used in each experiment. *, p<0.05.

Chronic diabetic conditions generally lead to kidney injury [28]. To test whether urinary miR-10a and miR-30d can be biomarkers for diabetes-induced renal injury, we employed streptozotocin (STZ)-treated diabetic mice as another kidney injury model. In this experiment, type 1 diabetes was induced in C57BL/6J mice with STZ treatment. In specific, mice were fasted for 4 h, followed by intraperitoneal injection with STZ at a dose of 50 mg/kg body weight for five days consecutively. Blood-glucose level test showed that STZ-treated mice maintained a hyperglycemia condition. Compared to the normal mice, the levels of kidney weight, blood glucose and 24 h urinary protein were all significantly increased in 2 month STZ-treated mice (Table 2). As shown in Figure 3, the urinary levels of miR-10a and miR-30d were significantly higher in STZ-treated mice compared with those in vehicle-treated control mice. In contrast, compared with the normal mice, the mice with only hyperglycemia showed either moderately decreased or unchanged levels of miR-10a and miR-30d in serum. Although the 24 h urinary protein level was not significantly increased after one month treatment with STZ, ultrastructural analysis indicated that, comparing to normal distribution of podocyte foot process, diabetic mice with one month STZ treatment showed apparent segmental podocyte foot process effacement (Figure 3E, arrow). These results suggest that the elevation of urinary miR-10a and miR-30d levels may specifically reflect hyperglycemia-induced kidney injury.

**Table 2:** Mouse kidney weight index, blood glucose and 24 h proteinuria (Mean ± SEM, n = 14).

	Kidney/Body weight(mg·g^−1^)	Blood glucose(mmol·L^−1^)	24 h proteinuria(mg)
Control	9.862±1.034	6.488±0.568	0.290±0.141
STZ (1 month)	11.463±1.165*	26.321±5.304**	0.541±0.276
STZ (2 months)	13.025±1.094*	27.274±4.841**	3.274±0.604**

Compared to the control group,

* *p*<0.05,

** *p*<0.01.

### Elevation of miR-10a and miR-30d levels in the urine of FSGS patients

**Figure 4:**
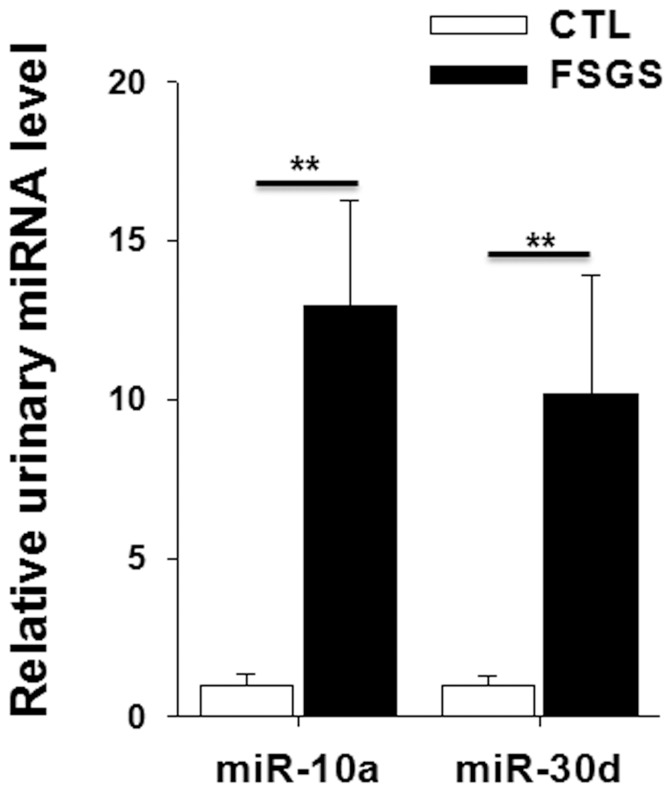
Alteration of the urinary miR-10a and miR-30d levels in FSGS patients. Urine samples were collected from 16 healthy donors and 16 FSGS patients. Note that, compared with healthy donors, FSGS patients had strikingly elevated levels of urinary miR-10a and miR-30d. **, p<0.01.

To find out whether the elevation of urinary miR-10a and miR-30d also occurs in patient with kidney injuries, we assessed the levels of urinary miR-10a and miR-30d in FSGS patients. Renal dysfunction of FSGS patients was also monitored by measuring blood pressure, BUN, serum creatinine, uric acid, urinary protein, urinary red blood cell, serum creatinine, creatinine clearance, triglyceride and total cholesterol levels. As shown in Table 1, FSGS patients had a significantly higher proteinuria level that the healthy controls. The levels of triglyceride and total cholesterol levels in these patients were also higher than those in the healthy controls. Next we compared the levels of miR-10a and miR-30d in urine from healthy volunteers with those from FSGS patients. As shown in Figure 4, compared with the levels of urinary miR-10a and miR-30d from healthy donors, the urine from FSGS patients contained about 13- and 10-fold higher concentrations of miR-10a and miR-30d, respectively. High levels of urinary kidney-enriched miR-10a and miR-30d clearly indicate the kidney injuries in FSGS patients.

## Discussion

Urine is regarded as a source for biomarkers, given its easy availability and reduced complexity when compared with serum. Many urinary proteins, such as neutrophil gelatinase-associated lipocalin (NGAL) [27], N-acetyl-β-D-glucosaminidase (NAG) [29], and ectodomain of kidney injury molecule-1 (Kim-1) [5], have been evaluated as noninvasive indicators of renal injury. However, problems with the reliable use of these proteins to identify and monitor kidney injury, including instability in the urine, modification due to physicochemical properties of the urine, delayed appearance, inconsistency of upregulation with different models of nephrotoxicity, and the absence of sustained elevation throughout the time course of renal injury to monitor the progression and regression of injury, limit the wide application of these marker proteins. As endogenous small noncoding RNAs, miRNAs hold great promise as unique accessible biomarkers to monitor tissue injury because of their size, abundance, tissue specificity, and relative stability. Indeed, accumulating evidence has demonstrated the usage of circulating miRNAs in various body fluids as an indicator for tissue injury [17], [20]. In the present study, we observed increases in the urinary concentrations of miR-10a and miR-30d corresponding to kidney injuries. Furthermore, urinary miR-10a and miR-30d exhibited a diagnostic sensitivity that was considerably superior to that of BUN when the results were correlated to the histopathological results.

### Kidney-enriched miRNAs are readily detected in human and animal urine

To serve as a biomarker, urinary miRNAs must be present at a considerable concentration to be readily detected. Our study clearly demonstrated that urinary miRNAs are stable and can be reliably extracted and assayed by qRT-PCR. By comparing the levels of miRNA in sera and urine, we found that kidney-enriched miRNAs, such as miR-10a and miR-30d, were present in urine, and their concentrations were approximately 1/10 of those in sera. Importantly, when kidney injury occurred, the levels of miR-10a and miR-30d in urine were strikingly elevated, while their levels in the serum were not increased. These results strongly suggest that urinary miR-10a and miR-30d can serve as ideal biomarkers for kidney injury. We used both I/R-induced acute kidney injury and STZ diabetes-induced chronic kidney injury animal models and showed that changes in the levels of urinary miR-10a and miR-30d occurred as a result of renal damage. Therefore, the relative concentration of kidney-enriched miRNA species has a potential to become a sensitive indicator for detecting and monitoring kidney injury (Figure 2).

Because miR-10a and miR-30d are enriched in kidney tissue (Figure 1), urinary miR-10a and miR-30d are probably derived directly from the kidneys, particularly when kidney injury has occurred. The recent work by Godwin et al. [11] showed that the expression profile of kidney miRNAs was significantly changed following renal ischemia-reperfusion injury (IRI). It is reasonable to hypothesize that certain upregulated miRNAs in the kidney may be released into the urine after IRI. Leakage of miRNAs from damaged cells or tissue has been reported previously [14]. However, it could also be true that renal cells and tissues actively release more miR-10a and miR-30d into circulation under the stress. Recent studies by our group and others have shown that, in response to various pathophysiological stimulations, cells can actively package miRNA into microvesicles and release them into circulation [30]. The high stability of urinary miRNAs suggests that they may be primarily encapsulated in kidney cell-secreted microvesicles.

### Urinary miRNAs are sensitive and specific indicators for kidney injury

We demonstrated using well-established models that kidney injuries can be precisely detected and monitored using a small number of circulating miRNA species. In our study, this miRNA-based method was more sensitive and probably more reliable than the current BUN method for detecting kidney injury. Elevation of the urinary miR-10a and miR-30d levels can be detected in mice with unilateral I/R in which the protein levels were not changed, suggesting that the urinary miR-10a and miR-30d levels can reflect mild or early kidney injury. In addition, urinary miR-10a and miR-30d are highly enriched to the kidney; therefore, the elevation of these miRNAs may be directly linked to the injuries of kidney. This hypothesis is supported by our observation that the elevation of miR-10a and miR-30d concentrations occurred only in urine and not in serum when mice were treated with renal I/R. Interestingly, recent study by Lorenzen et al. [31] showed that miR-10a was released into urine and could be detected in kidney transplant patients at the onset of acute T-cell mediated rejection, which may suggest a mild acute kidney injury during the process of T-cell mediated rejection.

The role of tissue miR-10a and miR-30d in kidney function also strengthens our conclusion that urinary miR-10a and miR-30d can serve as indicators for kidney injury. Previous studies have shown that miR-10a can target IL-12/IL-23p40 expression [32] and pro-apoptotic protein Bim [33], while miR-30d can negatively regulate apoptotic caspase CASP3 [34] and tumor suppressor p53 gene [35]. Through decreasing the levels of these apoptotic or pro-apoptotic proteins and inflammatory cytokines, miR-10a and miR-30d might provide a protection to kidney tissues/cells. In contrast, reduction of miR-10a and miR-30d in kidney cells would cause cell apoptosis and damage, which may finally lead to renal dysfunction. Indeed, it has been reported that during nephrogenesis, nephron progenitors highly expressing miR-10a and miR-10a can target Bim [24]. The study by Shi et al. [8] demonstrated that podocytes strongly expressed four members of the miR-30 family that may target genes such as vimentin, heat-shock protein 20 and immediate early response 3. Through the silencing of these target genes, the miR-30 and miR-10 miRNA families play an essential role in podocyte homeostasis and podocytopathies, which is in agreement with our finding in the present study. By measuring the levels of miR-10a and miR-30d in mouse urine (Figure 2) and mouse kidney tissue (Figure S3A) before and after kidney injury, and comparing the levels of pre-miR-10a and pre-miR-30d in kidney before and after kidney injury (Figure S3B), we found that during kidney injury, elevation of urinary miR-10a and miR-30d levels might be due to the leakage of miRNAs from the injured or damaged kidney cells. In other words, the elevation of miRNAs in urine is correlated to the reduction of miRNAs in kidney organs. Therefore, an elevation of urinary miR-10a/miR-30d levels correlates to a decrease of kidney miR-10a/miR-30d levels, which links to cell apoptosis and kidney injury/damage. Serving as negative regulators of cell apoptosis, miR-10a and miR-30d have been found to be upregulated in various cancer tissues, such as prostate cancer [36]. Clarifying the role of miR-10a and miR-30d in the tumorigenesis processes of these cancer cells may be helpful for understanding the correlation between urinary miR-10a/miR-30d and kidney injures.

Interestingly, although the chronic hyperglycemia caused an elevation of urinary miR-10a and miR-30d likely due to the kidney damage, a short period of high blood glucose exposure did not increase the level of these kidney-specific miRNAs in urine. By challenging 12 h–fasting mice with an intraperitoneal injection of glucose (2 g/kg of body weight), we found no elevation of urinary miR-10a and miR-30d within 1–3 h (data not shown). These results may suggest that the increase in urine miRNA levels in the diabetes mouse model is not due to a glycosuria-induced osmotic diuresis.

The results for the human urine samples further confirmed the feasibility of using the urinary miR-10a and miR-30d levels to detect kidney injury in humans. As shown in Figure 4, we found that the urinary miR-10a and miR-30d levels in FSGS patients were significantly higher than those in healthy volunteers, indicating the severity of the kidney injuries in these patients. In summary, our study demonstrated that miR-10 and miR-30d are stably present in human and animal urine and that the elevation of the urinary miR-10a and miR-30d levels can serve as a novel urine-based biomarker of kidney injury.

## Supporting Information

Figure S1 **Relative levels of KIM-1 and NGAL gene expression in mouse kidney with/without renal ischemia-reperfusion injury.** The levels of KIM-1 and NGAL mRNA was assayed by qRT-PCR. The data are presented as means ± SEM for three independent experiments; three mice were used in each experiment.
(TIF)

Figure S2 **Level of serum miR-10a and miR-30d in mice with/without renal ischemia-reperfusion injury.** **A**, the serum miR-10a level was decreased in DS I/R mice but not SS I/R mice. **B**, no alteration in the serum miR-30d level in mice with either SS or DS I/R treatment was observed. The data are presented as means ± SEM deviation for six independent experiments; three mice were used in each experiment. *, p<0.05.
(TIF)

Figure S3 **The levels of miR-10a, miR-30d, pre-miR-10a and pre-miR-30d in mouse kidney tissues detected by TaqMan probe-based qRT-PCR with U6 serving as an internal control.** A) Levels of miR-10a and miR-30d in mouse kidney with or without renal I/R. B) Levels of pre-miR-10a and pre-miR-30d in mouse kidney with or without renal I/R. Note that, following renal I/R, the levels of mouse kidney miR-10a and miR-30d are decreased whereas the levels of pre-miR-10a and pre-miR-30d are not changed. The data are presented as means ± SEM for three independent experiments. *, p<0.05. **, p<0.01.
(TIF)

Table S1 Relative enrichment of miRNAs in various mouse tissues detected by Solexa sequencing. Nine mouse organs including kidney, liver, spleen, brain, intestine, lung, etc. were assayed. After normalization, the miRNAs with total counts from in nine mouse organs >2000 were selected. The ratio of miRNAs in other tissues such as lung, brain, etc. was not shown due to the limited space.
(DOC)
