# Supplementary figures and images for: Urinary MicroRNA-10a and MicroRNA-30d Serve as Novel, Sensitive and Specific Biomarkers for Kidney Injury
Source: PLoS One. 2012 Dec 13;7(12):e51140. doi: 10.1371/journal.pone.0051140 (PMC3521774; doi:10.1371/journal.pone.0051140)

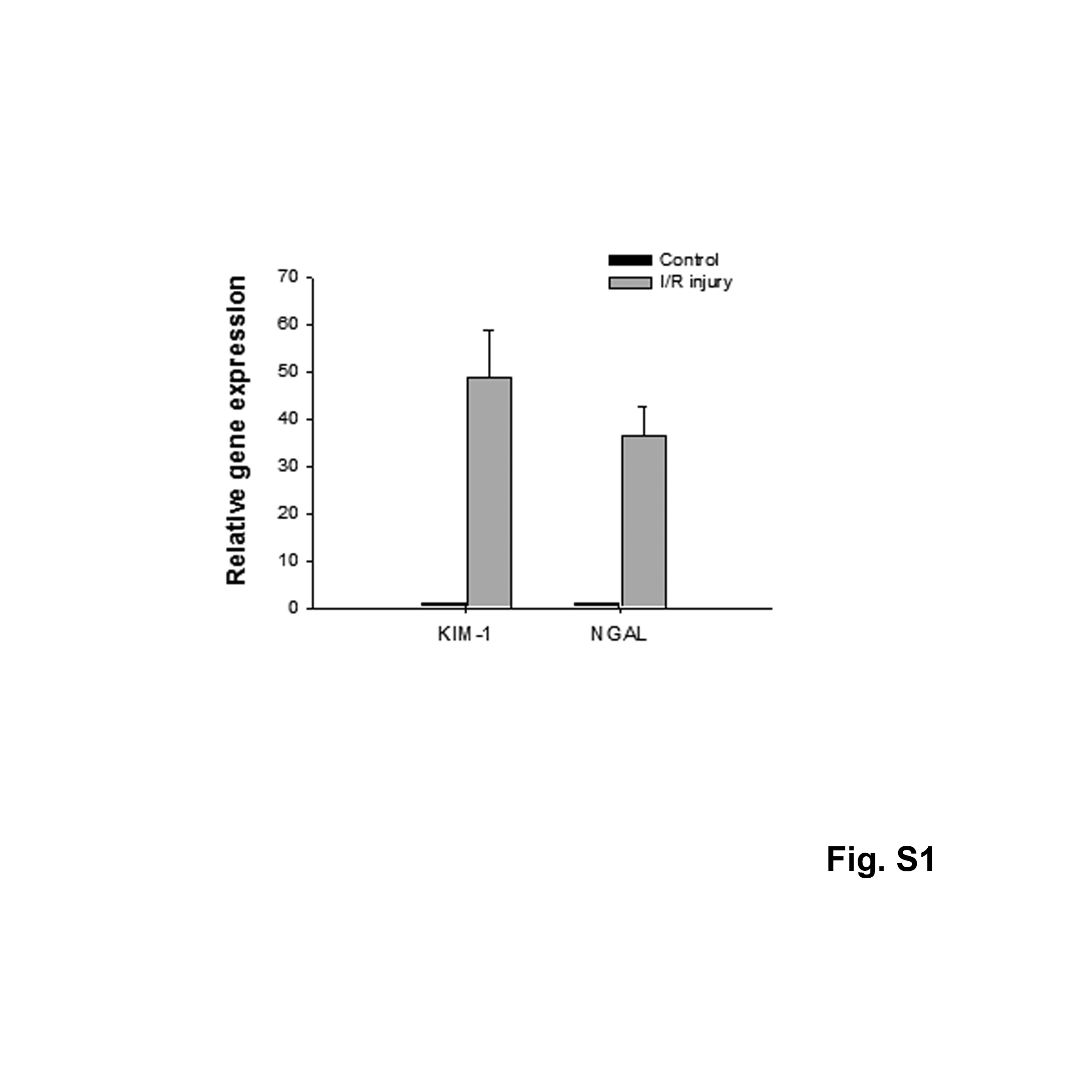

Supplement: Figure S1 — Relative levels of KIM-1 and NGAL gene expression in mouse kidney with/without renal ischemia-reperfusion injury. The levels of KIM-1 and NGAL mRNA was assayed by qRT-PCR. The data are presented as means ± SEM for three independent experiments; three mice were used in each experiment. (TIF) [file pone.0051140.s001.tif]

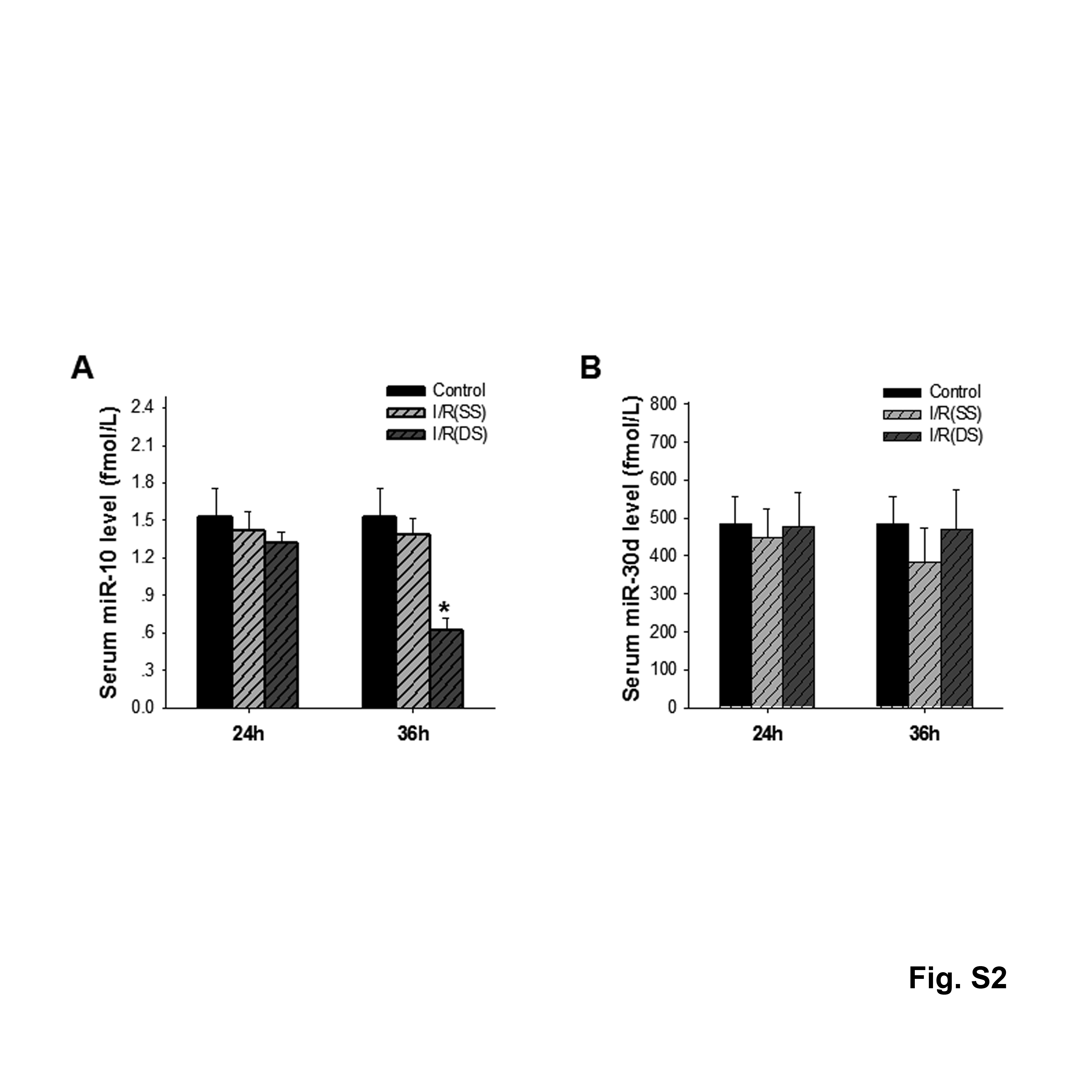

Supplement: Figure S2 — Level of serum miR-10a and miR-30d in mice with/without renal ischemia-reperfusion injury. A, the serum miR-10a level was decreased in DS I/R mice but not SS I/R mice. B, no alteration in the serum miR-30d level in mice with either SS or DS I/R treatment was observed. The data are presented as means ± SEM deviation for six independent experiments; three mice were used in each experiment. *, p<0.05. (TIF) [file pone.0051140.s002.tif]

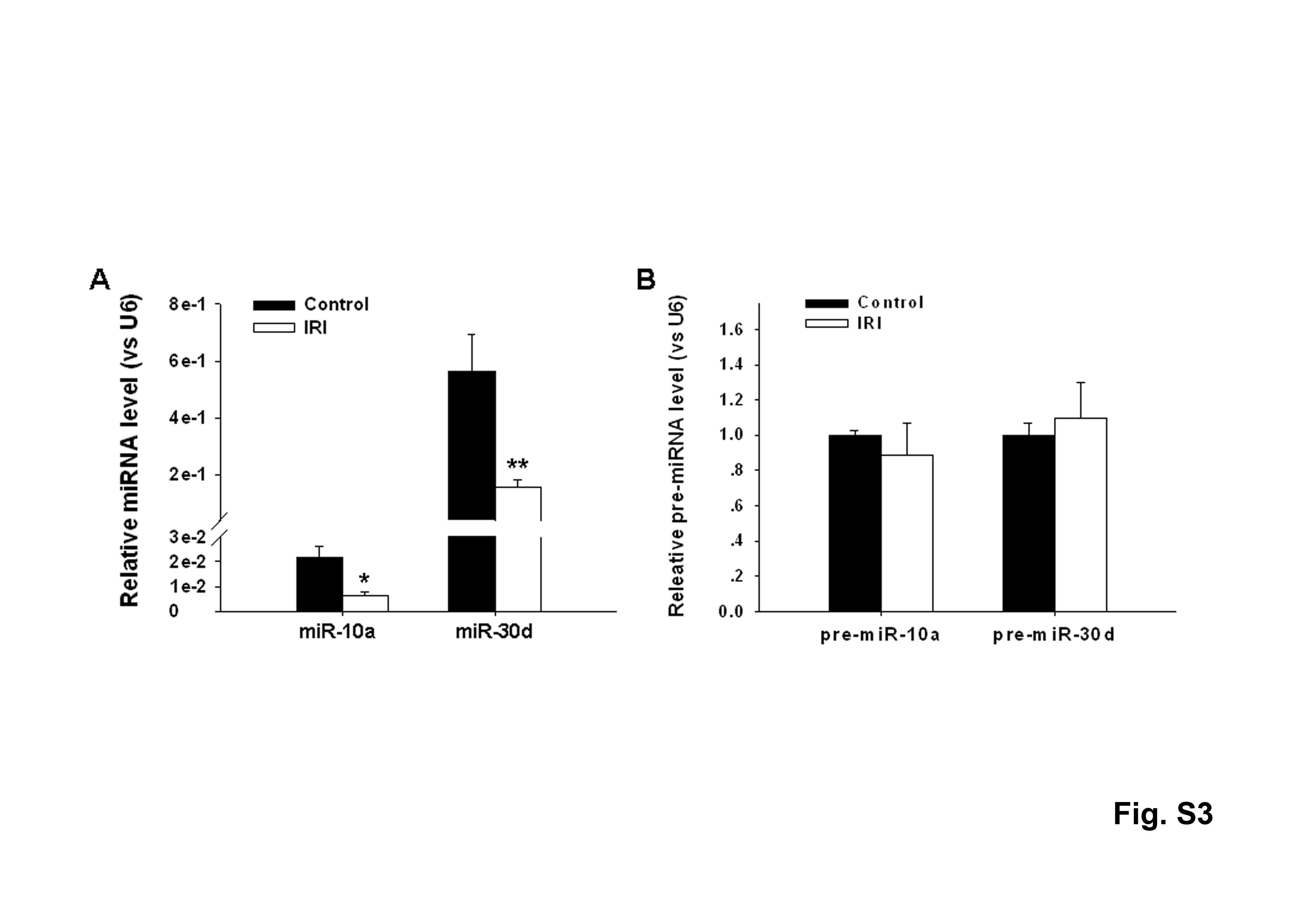

Supplement: Figure S3 — The levels of miR-10a, miR-30d, pre-miR-10a and pre-miR-30d in mouse kidney tissues detected by TaqMan probe-based qRT-PCR with U6 serving as an internal control. A) Levels of miR-10a and miR-30d in mouse kidney with or without renal I/R. B) Levels of pre-miR-10a and pre-miR-30d in mouse kidney with or without renal I/R. Note that, following renal I/R, the levels of mouse kidney miR-10a and miR-30d are decreased whereas the levels of pre-miR-10a and pre-miR-30d are not changed. The data are presented as means ± SEM for three independent experiments. *, p<0.05. **, p<0.01. (TIF) [file pone.0051140.s003.tif]
