# Supplementary material for: Urinary MicroRNA-10a and MicroRNA-30d Serve as Novel, Sensitive and Specific Biomarkers for Kidney Injury
Source: PLoS One. 2012 Dec 13;7(12):e51140. doi: 10.1371/journal.pone.0051140 (PMC3521774; doi:10.1371/journal.pone.0051140)
Supplement: Table S1 — Relative enrichment of miRNAs in various mouse tissues detected by Solexa sequencing. Nine mouse organs including kidney, liver, spleen, brain, intestine, lung, etc. were assayed. After normalization, the miRNAs with total counts from in nine mouse organs >2000 were selected. The ratio of miRNAs in other tissues such as lung, brain, etc. was not shown due to the limited space. (DOC) [file pone.0051140.s004.doc]

**Supplemental Table S1.** Relative enrichment of miRNAs in various mouse tissues detected by Solexa sequencing. Nine mouse organs including kidney, liver, spleen, brain, intestine, lung, etc. were assayed. After normalization, the miRNAs with total counts from in nine mouse organs > 2000 were selected. The ratio of miRNAs in other tissues such as lung, brain, etc. was not shown due to the limited space.

| **miRNA name** | **Kidney** | **Liver** | **Spleen** |
| --- | --- | --- | --- |
| mmu-miR-30d | 56% | 0% | 0% |
| mmu-miR-10a | 52% | 0% | 1% |
| mmu-miR-196b | 50% | 0% | 1% |
| mmu-miR-30c | 46% | 1% | 6% |
| mmu-miR-30e | 44% | 1% | 7% |
| mmu-miR-196a | 41% | 1% | 3% |
| mmu-miR-30a | 38% | 0% | 0% |
| mmu-miR-22 | 36% | 3% | 5% |
| mmu-miR-185 | 33% | 1% | 10% |
| mmu-miR-10a* | 32% | 1% | 3% |
| mmu-miR-429 | 31% | 2% | 2% |
| mmu-miR-200a | 27% | 0% | 0% |
| mmu-miR-30e* | 25% | 1% | 21% |
| mmu-miR-27b | 25% | 2% | 3% |
| mmu-miR-140* | 25% | 2% | 21% |
| mmu-miR-101a | 25% | 1% | 10% |
| mmu-miR-33 | 24% | 2% | 12% |
| mmu-miR-29c | 24% | 1% | 3% |
| mmu-miR-15a | 24% | 3% | 20% |
| mmu-miR-30e* | 24% | 1% | 11% |
| mmu-miR-423-5p | 23% | 2% | 4% |
| mmu-miR-30c-2* | 23% | 4% | 2% |
| mmu-miR-200b | 22% | 0% | 0% |
| mmu-miR-30a* | 22% | 2% | 4% |
| mmu-miR-872 | 22% | 1% | 7% |
| mmu-miR-148b | 21% | 2% | 6% |
| mmu-miR-30b | 21% | 1% | 5% |
| mmu-miR-340-5p | 20% | 2% | 16% |
| mmu-miR-744 | 19% | 0% | 0% |
| mmu-miR-378 | 19% | 2% | 3% |
| mmu-miR-542-3p | 19% | 1% | 4% |
| mmu-miR-29a | 18% | 1% | 6% |
| mmu-miR-200b* | 18% | 0% | 0% |
| mmu-miR-24 | 18% | 1% | 8% |
| mmu-miR-191 | 18% | 1% | 20% |
| mmu-miR-451 | 18% | 1% | 37% |
| mmu-miR-106b | 17% | 1% | 30 |
| mmu-miR-29b | 16% | 1% | 8% |
| mmu-miR-130a | 16% | 1% | 12% |
